# Analysis of Fiber-Reinforced Concrete Slabs under Centric and Eccentric Load

**DOI:** 10.3390/ma14237152

**Published:** 2021-11-24

**Authors:** Zuzana Marcalikova, Vlastimil Bilek, Oldrich Sucharda, Radim Cajka

**Affiliations:** 1 Department of Structures, Faculty of Civil Engineering, VSB-Technical University of Ostrava, Ludvíka Podéště 1875/17, 708 00 Ostrava, Czech Republic; radim.cajka@vsb.cz; 2 Department of Building Materials and Diagnostics of Structures, Faculty of Civil Engineering, VSB-Technical University of Ostrava, Ludvíka Podéště 1875/17, 708 00 Ostrava, Czech Republic; vlastimil.bilek@vsb.cz (V.B.); oldrich.sucharda@vsb.cz (O.S.)

**Keywords:** soil-structure interaction, fibers, fiber-reinforced concrete, slab, subsoil, experiments, mechanical properties

## Abstract

Research on the interaction between slabs and subsoil involves the field of materials engineering, concrete structures, and geotechnics. In the vast majority of cases, research focuses on only one of these areas, whereas for advanced study and computer simulations, detailed knowledge of the whole task is required. Among the new knowledge and information upon which this article focuses is the evaluation of subsoil stress using specialized pressure cells, along with detailed measurements of the deformation of a fiber-reinforced concrete slab. From a design point of view, this research is focused on the issue of the center of the cross section and the influence of eccentricity. Knowledge in this area is not yet comprehensively available for fiber-reinforced concrete slabs, where 2D deformation sections of the slab and 3D deformation surfaces of the slab are used in experiments. The experimental program includes a centrically and eccentrically loaded slab. These are structural elements that were tested on a specialized device. Both slabs had the same concrete recipe, with a dispersed reinforcement content of 25 kg/m^3^. The dimensions of the slab were 2000 × 2000 × 150 mm. Laboratory tests assessed compressive strength, the modulus of elasticity, splitting tensile strength, and bending tensile strength. Based on approximate data from the 3D deformation surfaces, an evaluation of the load-displacement diagrams for the center of the slab and for the center of eccentricity was performed. In conclusion, an overall evaluation and discussion of the results relies on experiments and the mechanical properties of fiber-reinforced concrete.

## 1. Introduction

One of the most used building materials is concrete [1,2]. However, current design codes and recommendations focus mainly on its basic mechanical properties. These include good compressive strength [3], modulus of elasticity, and, to a limited extent, tensile strength. However, more detailed analysis requires a comprehensive description based, for example, on the use of fracture mechanics [4]. Optimization of a specific concrete recipe is also often addressed [5,6]. Unfortunately, low tensile strength is a characteristic of concrete [7]. For this reason, one must reinforce concrete [8,9]. Reinforced concrete has significantly better tensile strength due to the use of steel and other materials [10,11]. However, the major disadvantage of reinforced concrete structures is the hard work required to make them, as it is necessary to ensure the correct position of the concrete reinforcement, sufficient coverage of the concrete reinforcement, the interaction of the concrete reinforcement and the concrete, and the requirements that arise for the reinforcement bond.

A variant of concrete where these specifics disappear is so-called fiber-reinforced concrete [12,13,14,15]. By inserting dispersed reinforcement into concrete, tensile strength is increased [13,16,17,18,19]. It is possible to choose both the size of the fiber dosing and the type/shape of the fibers themselves, which is important to ensure cohesion between concrete and dispersed reinforcement, where the shape of the fiber itself also has a significant effect on the residual tensile strength of concrete and a shape load–displacement diagram [12,13,14,15,16,17,18,19]. Inverse analysis and identification [20,21] can be used for a comprehensive description of the mechanical properties of fiber-reinforced concrete. Steel fiber can also have an effect on concrete abrasion resistance [22].

**Figure 1:**
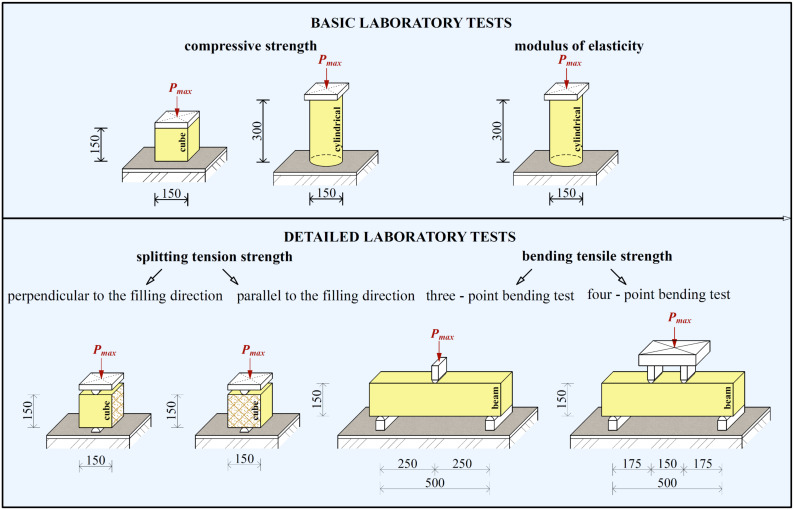
Set of laboratory tests performed.

From the abovementioned works, the significant potential of fiber-reinforced concrete as a construction material for load-bearing structures is evident [12,13,14,15,16,17,18,19,20,21]. However, the many fiber-reinforced concrete variants that can be produced complicates its application. A suitable application often leads to an individual fiber-reinforced concrete recipe. The remaining issue is with the determination of a comprehensive description of the mechanical properties. There are a number of test variants, especially for determining tensile strength. With regard to the analyzed and presented works, a comprehensive experimental laboratory program was designed for the experimental program in Figure 1, focused mainly on comparative tests of three- and four-point bending tests.

**Figure 2:**
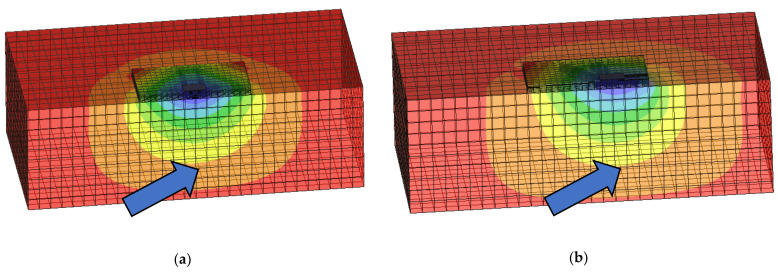
Deformations for slab: (**a**) centrically loaded slab; (**b**) eccentrically loaded slab.

**Figure 3:**
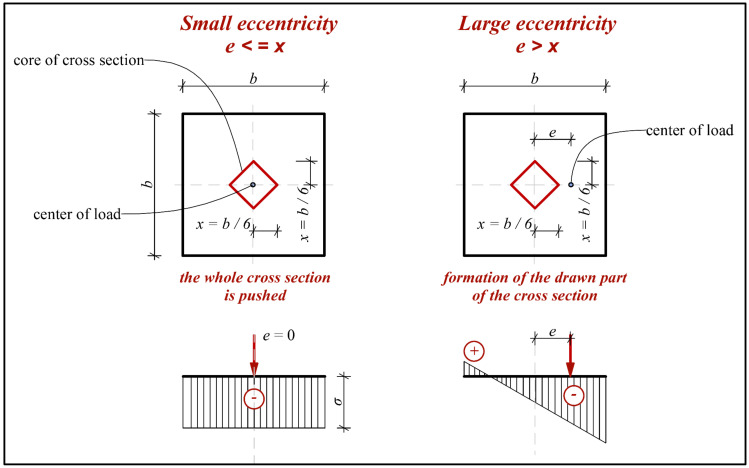
Core of cross section of slab (*e* = eccentricity; *b* = size of slab; *x* = the distance of the load to the center of the slab.

One of the possible uses of fiber-reinforced concrete in construction is in foundation structures and floors [23,24,25,26]. Very interesting experiments in [23] deal in detail with selected tests of slabs and their collapse. However, slab tests are only performed with a limited laboratory simulation of the subsoil, when it is not possible, for example, to determine the active depth in more detail. The laboratory program for mechanical properties is also performed in the basic scope. One interesting experiment with slabs in interaction with subsoil is presented in [24]. The tests were performed for dosages of 20 and 30 kg/m^3^. The experiments in [23,24,25,26] had a greater number of test variants, but were not focused on eccentric loading or monitoring of subsoil stress. The effect of the eccentric load on the slab in interaction with the subsoil is illustrated in Figure 2. The course of deformations is very different. The difference between the slab with centric and eccentric loads is also evident from Figure 3. Figure 3 also shows the size of the pressed and drawn cross-sectional area.

Therefore, part of this research focuses on the laboratory testing of fiber-reinforced concrete to determine its mechanical properties, while another part of the research focuses on the experimental testing of fiber-reinforced concrete slabs to evaluate their behavior when exposed to the environmental effects of loading. This research builds on previous experiments on slabs in interaction with subsoil using numerical modeling [27,28]. The newly selected and presented research plan was conceived for significantly different subsoil, and the effect of load eccentricity on the overall course of deformations was also tested. It is also very important to expand the measurement and evaluation of specialized pressure cells in the subsoil.

In the case of solved tasks, it is necessary to take into account, among other things, the influence of the subsoil itself and the interactions between the foundation and the subsoil and between the structure and the foundation; we call such a task SSI (soil–structure interaction). For this reason, it is also very important to determine the type of subsoil, e.g., by using a geological profile, where the individual subsoil layers and their mechanical properties, such as stiffness (rigid or flexible subsoil) and cohesion, are visible [29]. It is good to know the mechanical properties of the subsoil to a depth where the significant effect of external loads is evident, often referred to as the depth of the deformation zone. In case of unsuitable subsoil, it is possible to replace the subsoil via so-called subsoil homogenization. Determining the stiffness of the foundation also affects the overall behavior and interaction. Much attention is given to said area of slab experiments in interaction with the subsoil [30,31,32,33,34,35,36,37] and to numerical modeling [38,39]. However, detailed numerical modeling based on a non-linear analysis requires a comprehensive description of mechanical properties and the execution of full-scale experiments so that the results are of sufficient value. However, a large number of the listed experiments are devoted to concrete or reinforced concrete, and the behavior of fiber-reinforced concrete is significantly different than that of non-reinforced concrete.

The methodology and experimental program of research area were determined on the basis of current design-code approaches in the field of geotechnics [40] and concrete structures, which are based on the recommendations of the Model Code 1990 [1]. The Model Code 2010 [2] recommendation was also created by the wider application of concrete and its new variants. The summary information was used to design experiments that focused on the overall understanding of the problem of slab interaction with the subsoil. The test-specialized loading equipment Stand was designed especially for the resolution of the mentioned problem [41].

The presented research, as already mentioned, deals with the determination of the mechanical properties of fiber-reinforced concrete on the basis of laboratory testing and experimental testing of fiber-reinforced concrete slabs. Basic tests to determine the compressive strength and modulus of elasticity, as well as detailed tests to determine tensile strength, have been performed [42,43,44,45]. The set of basic and specialized tests is shown in Figure 1. The mechanical properties of concrete are also addressed [46,47,48,49,50,51]. However, in the case of fiber-reinforced concrete, it is more appropriate to perform our own specialized tests, where the above recommendations and standards cannot simply be modified for fiber-reinforced concrete.

**Figure 4:**
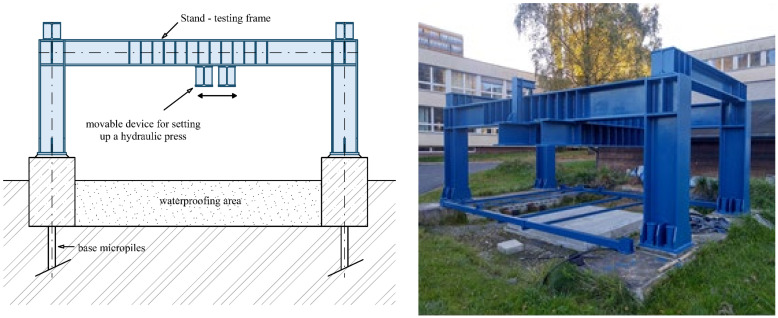
Specialized loading equipment Stand.

Experimental testing of fiber-reinforced concrete slabs was carried out at the Faculty of Civil Engineering, VSB—Technical University of Ostrava, Czech Republic, where there is specialized loading equipment called Stand (Figure 4). It is possible to apply have a load of up to 1000 kN tested by the equipment, and it is connected to a universal bus system for measuring deformations and load forces. It is also possible to change the position of the slab load arbitrarily in one direction by means of a movable device mounted on the stand and a hydraulic press.

## 2. Experimental Program

**Figure 5:**
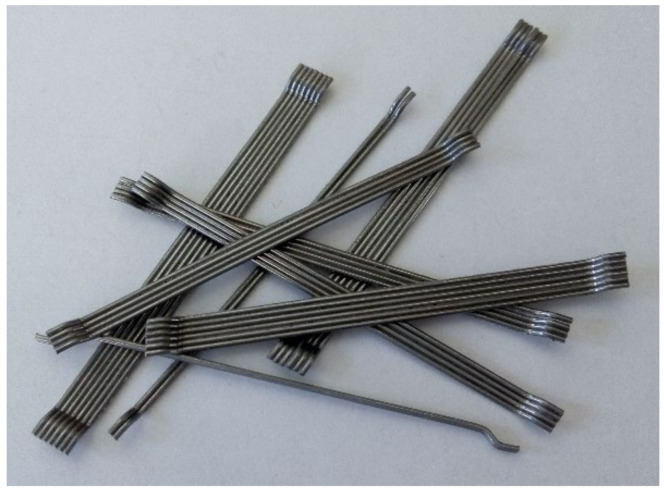
Fiber Dramix^®^ 3D 65/60 BG.

Transport concrete was used for the production of slabs and laboratory samples, where the recipe and the content of individual components in the concrete mixture are given in Table 1. The concrete was reinforced with Dramix^®^ 3D 65/60 BG fibers [52] with a dosage of 25 kg/m^3^. The basic mechanical characteristics of the fibers are given in Table 2. The fibers have so-called end bends to ensure better cohesion with the concrete. The fibers are shown in Figure 5.

The laboratory program included tests of samples to determine the compressive strength, modulus of elasticity, splitting tensile strength, and bending tensile strength [42,43,44,45]. Compressive strength was determined on cubes (*f_c, cube_*) measuring 150 × 150 × 150 mm and on cylinders (*f_c, cyl_*) with a diameter of 50 mm and a height of 300 mm. The test scheme is shown in Figure 1.

Very important properties also include tensile strength, which was determined by the splitting tensile test and the bending tensile strength test. Two sample loading variants were selected for the split tensile test. The first variant was for testing the splitting tensile strength perpendicular to the direction of filling. The second variant was for testing the splitting tensile strength parallel to the direction of filling. The schema is shown in Figure 1. The splitting tensile strength was then determined according to the following equation [44]:

(1) $$f_{ct,sp}=\frac{2\cdot F}{\pi \cdot l\cdot d},$$

where *F* is the maximum load at failure of the sample, *l* is the length of the line of contact, and *d* is the transverse dimension of the body. 

An alternative for determining tensile strength is a bending tensile test. The scheme and dimensions of the samples for both types of tests are shown in Figure 1. The span between the supports was 500 mm for both types of tests. Bending tensile strength can be determined according to the following equations:

- Three-point bending test [43]:
  (2) $$f_{c,fl,3B}=\frac{3\cdot F\cdot L}{2\cdot b\cdot h^{2}}$$
- Four-point bending test:
  (3) $$f_{c,fl,4B}=\frac{6\cdot F\cdot e}{2\cdot b\cdot h^{2}}$$
  where *F* is the maximum failure load of the sample, *L* is the span, *b* and *h* are the transverse dimensions of the specimen, and *e* is the distance between the support and the force.

**Figure 6:**
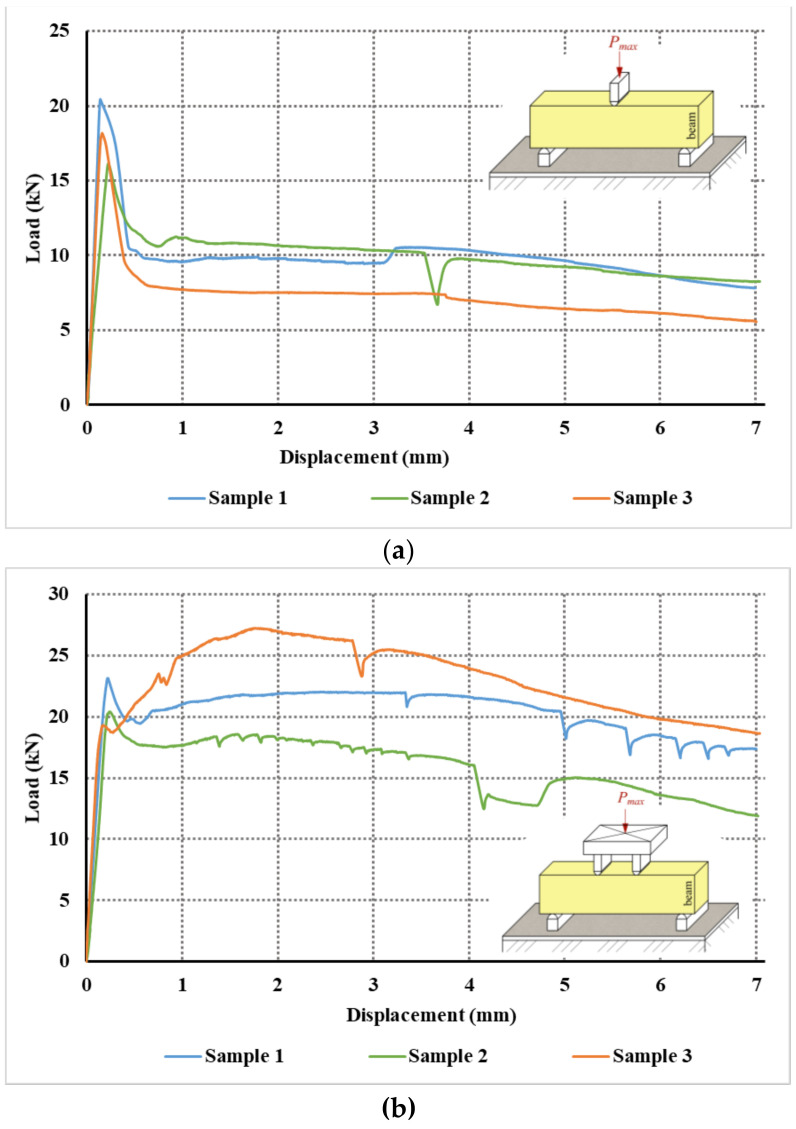
Load–displacement diagrams for: (**a**) three-point bending test; (**b**) four-point bending test.

From the test record, evaluations of the load–displacement diagrams for the three-point and four-point test were also performed; the diagrams are shown in Figure 6.

In the case of a three-point bending test, the load–displacement curves are very similar. After the initialization of the crack, the load will decrease significantly to about 50–60% of the maximum load. The load gradually decreases with the development and opening of the crack. In the test, the crack was close to the load, i.e., at the point of maximum bending moment. In the four-point bending test, the results differ. In one of the selected tests, there was even tensile strengthening. In a four-point test, the location of the crack is not predefined. A crack can and did form between the loading forces. Gradually, the load decreased, and a crack opened up and increased deformations.

Based on all laboratory tests, the mechanical properties of fiber-reinforced concrete were determined, with the average values given in Table 3. The average cubic compressive strength of concrete was 24 MPa and the average cylindrical compressive strength was 20.5 MPa. The conversion coefficient between the cylindrical compressive strength, *f_c, cyl_*, and the cubic compressive strength, *f_c, cube_*, can be expressed as a mutual ratio of their values. The average value of this coefficient is 0.85, where this is the usual value. The split tensile strength was approximately 1/10 of the cylindrical strength. This is also the usual ratio.

A comparison was performed by conversion to uniaxial tensile strength. It is also possible to determine the value of the uniaxial tensile strength from the splitting tensile test as follows:

(4) $$f_{ct}=f_{ct,sp}$$

or by conversion from a bending tensile test using the following equation:

(5) $$f_{ct}=\frac{f_{ct,fl}}{1.65}$$

**Figure 7:**
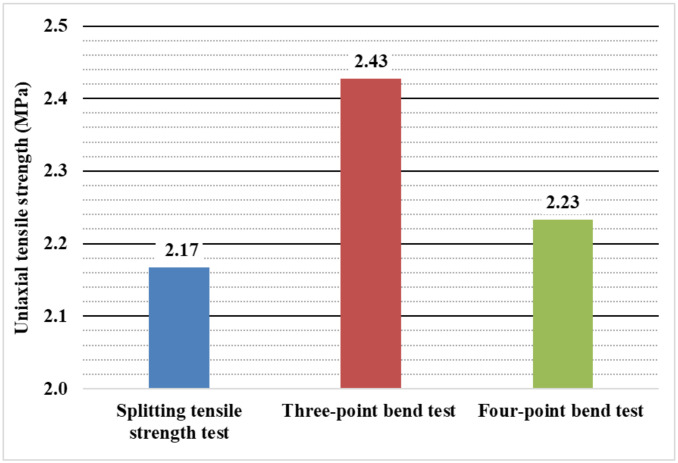
Uniaxial tensile strength for individual types of tests.

Figure 7 shows the recalculated values of uniaxial tensile strength. From the splitting tensile test, the value of uniaxial tensile strength was determined to be 2.17 MPa and 2.43 MPa and 2.23 MPa for the three- and four-point bending tests, respectively. The differences between the four-point test and the splitting tensile test are very small. On the other hand, for the three-point bending test, the tensile strengths are slightly higher.

**Figure 8:**
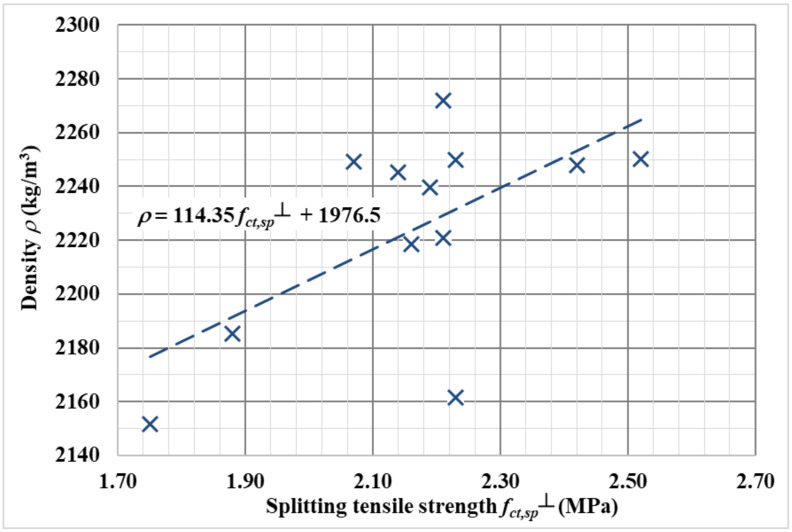
Functional dependence of splitting tensile strength (perpendicular to the filling direction) on density.

Furthermore, the functional dependence of the splitting tensile strength (perpendicular to the filling direction) and the bulk density was determined, as shown in Figure 8. The resulting functional dependence is:

(6) $$\rho =114.35\cdot f_{ct,sp}+1976.5.$$

It is clear from Figure 8 that with a higher bulk density of concrete, the split tensile strength also increases. It can be assumed that the bulk density is affected by the larger proportion of steel fibers.

## 3. Parameters of Subsoil

**Figure 9:**
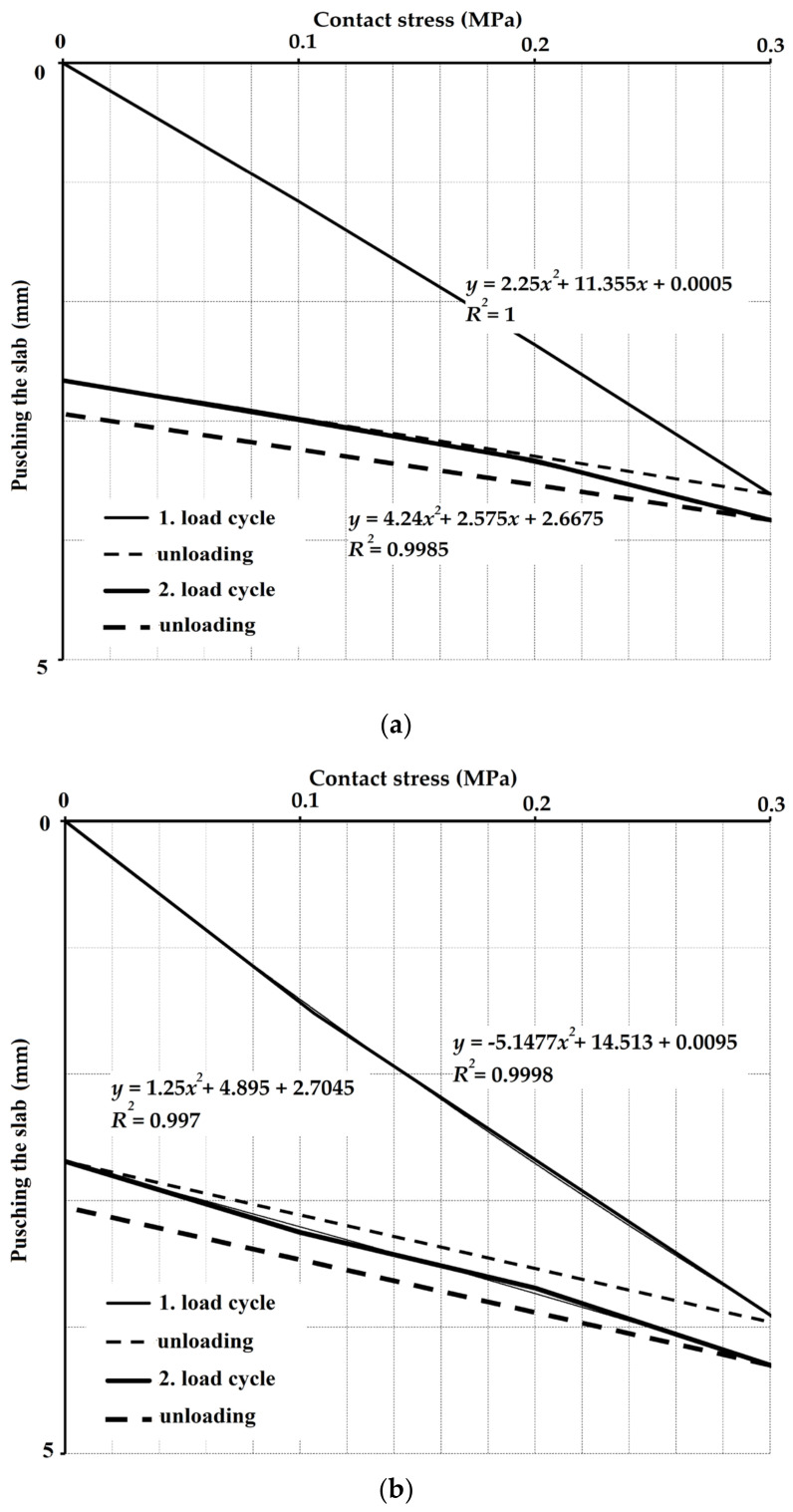
Dependence of pushing the slab on contact stress—hysteresis loops: (**a**) before concreting the centrically loaded slab G10, *e* = 1.50 m; (**b**) before concreting the centrically loaded slab G10, *e* = 0.75.

Among other things, the parameters of the subsoil are also very important in slab experiments. In tests of slabs in interaction with the subsoil, soil was used that, before compaction, had the character of incoherent soil (approx. 55% sand, 15% gravel, and 30% clay). This soil can be globally classified, according to the already invalid standard CSN 731001 [53], as clayey sand SC (S5). The subsoil also had the characteristics given in Table 4 and Table 5. The modulus of deformation of the subsoil was calculated on the basis of the following equation [54]:

(7) $$E_{def}=\frac{1.5\cdot r}{\left(a_{1}+a_{2}\cdot p_{max}\right)}$$

where *r* is the radius of the load plate (150 mm), *a*_1_ and *a*_2_ are the constants of the 2nd degree polynomial corresponding to the stress dependence of the plate loading (Figure 9), and *p_max_* is the maximum contact stress, i.e., 0.3 MPa.

When setting the modulus of deformation of the subsoil, it is appropriate to determine the *E_def,2_*/*E_def,1_* ratio. In the case of pre-experiment measurements, the ratio of modulus of deformation averaged 2.8. From the above, it can be assumed that the soil has a relatively large deformation capacity. The modulus of deformation ratio determined after the tests decreased to 2.1. The measurement results also show that *E_def,1_* is very similar before and after the tests and is in the range of 16–19 MPa. The differences for the modulus of deformation *E_def,2_* are more pronounced, in the range of 32–58 MPa.

To determine the physical and descriptive characteristics of the soil, two samples were taken at different places under the base slab. The strength characteristics were determined on a reconstituted 100% sample on a box shear device (sample size: 100 × 100 × 20 mm).

## 4. Testing Equipment and Measurement

**Figure 10:**
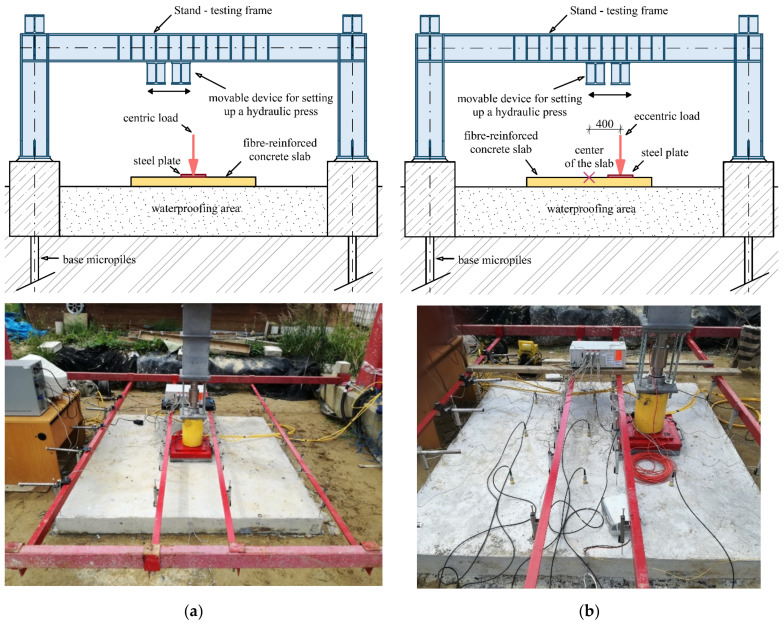
Slab: (**a**) centrically loaded slab G10; (**b**) eccentrically loaded slab G11.

The experimental tests consisted of two fiber-reinforced concrete slabs that differed in the position of the load. The first slab, G10, was loaded in its center, so it was a centrically loaded slab (Figure 10a).

**Figure 11:**
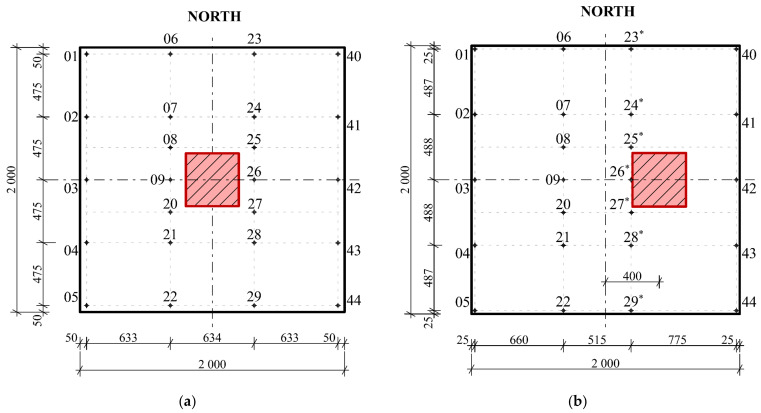
Sensor layout schema: (**a**) centrically loaded slab G10; (**b**) eccentrically loaded slab G11.

In the case of the eccentrically loaded slab G11, the position of the load was situated 400 mm from the center of the slab, i.e., it was an eccentrically loaded slab G11 (Figure 10b). Both fiber-reinforced concrete slabs had dimensions of 2000 × 2000 × 150 mm and were concreted into steel formwork. During the test, a constant increase in load was made possible by means of a hydraulic press. The loading of the slabs was performed in loading steps of 75 kN until complete collapse. Each loading step was performed after 30 min. To ensure load distribution, the hydraulic press was mounted on a steel plate measuring 400 × 400 mm. Sensor-layout schemata for a given type of slab are shown in Figure 11a,b. A total of 24 track sensors were installed.

## 5. Results of Experimental Tests of Slab

**Figure 12:**
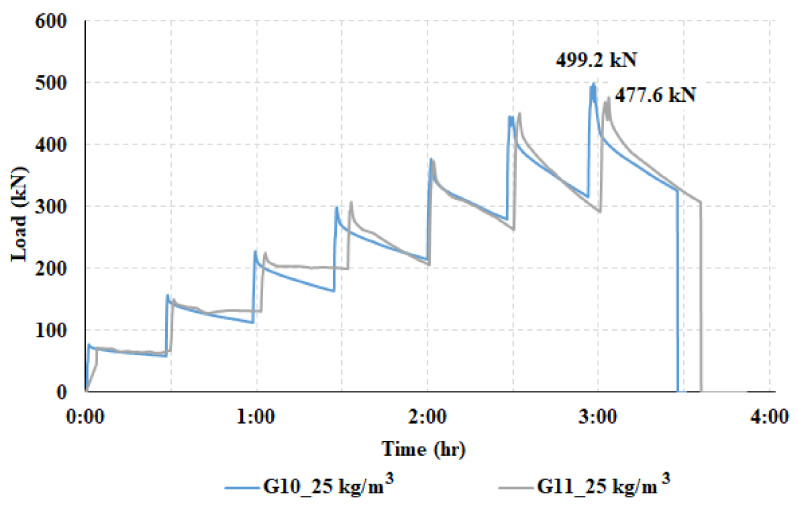
Loading in steps of the centrically loaded slab (G10) and eccentrically loaded slab (G11).

Based on the experimentally measured data, a diagram (Figure 12) was created showing the course of the test for a centrically (G10) and eccentrically loaded slab (G11). It can be seen from the diagram that the loading of both slabs was ended in the seventh loading step, when the pressure in the hydraulic press dropped, resulting in the destruction of both fiber-reinforced concrete slabs. The measurement of a centrically loaded slab (G10) was completed at a load of 499.2 kN, and for an eccentrically loaded slab (G11), at a load of 477.6 kN. The difference in the maximum achieved load value for both slabs is relatively small. It is clear from the graph that the relaxation of the slab is significantly smaller for the initial loading steps than for the loading before the end of the test. The decrease in the bending stiffness of the slab is also more pronounced when cracks in the fiber-reinforced concrete slab are already visible.

**Figure 13:**
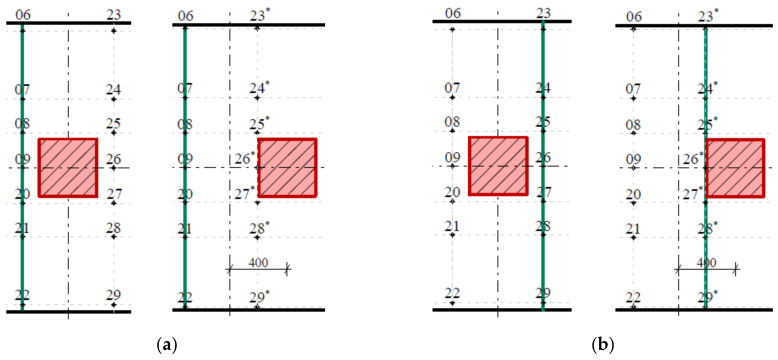
Monitored 2D sections: (**a**) first monitored 2D section for centrically loaded slab G10 (left) and eccentrically loaded slab G11 (right); (**b**) second monitored 2D section for centrically loaded slab G10 (left) and eccentrically loaded slab G11 (right).

The first part of the experimental measurement of the slabs was monitoring the deformation behavior of the slab in the transverse direction. In the framework of this article, the sections correspond to track sensors 06–22 (Figure 13a), 23|23*–29|29* (Figure 13b). From the data read by the track sensors, the deformations of the slab at the locations of the track sensors were calculated.

**Figure 14:**
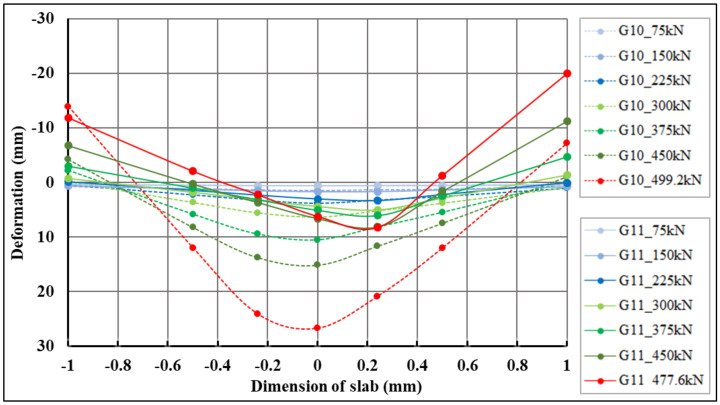
Comparison of 2D cross-deformation sections of centrically loaded slab G10 and eccentrically loaded slab G11 for sensors 06–22.

The maximum negative deformations in the slabs of track sensors 06–22, which correspond to the lifted corners of the slabs, measured 13.93 mm (centrically loaded slab G10, sensor 06) and 20.01 mm (eccentrically loaded slab G11, sensor 22), respectively. The maximum positive deformations in the slabs of track sensors 06–22, which correspond to the pushing of the slabs into the subsoil, were measured at 26.72 mm (centrically loaded slab G10, sensor 09) and 8.24 mm (eccentrically loaded slab, sensor 20), respectively. Comparative 2D deformation sections at the locations of track sensors 06–22, which capture the behavior of both fiber-reinforced concrete slabs, are shown in Figure 14. Deformation values for selected loading steps and selected sensors in section 06–22 for centrically (G10) and eccentrically (G11) loaded slabs are shown in Table 6. In the case of eccentrically loaded slab G11, the lift of the corners of the slab (negative deformation) at track sensor of 22 was approximately triple compared to centrically loaded slab G10. The pushing of the slab (positive deformation) of centrically loaded slab G10 was approximately quadruple at track sensor of 09 in comparison with eccentrically loaded slab G11, as shown in Figure 14 and Table 6.

The maximum negative deformations at the locations of track sensors 23|23*–29|29*, which correspond to the lifted corners of the slabs, were 8.93 mm (centrically loaded slab G10, sensor 23) and 10.57 mm (eccentrically loaded slab G11, sensor 29*), respectively. Maximum positive deformations at the locations of track sensors 23|23*–29|29*, which correspond to the pushing of the slabs into the subsoil, were 24.36 mm (centrically loaded slab G10, sensor 26) and 18.79 mm (eccentrically loaded slab G11, sensor 27*), respectively. 

**Figure 15:**
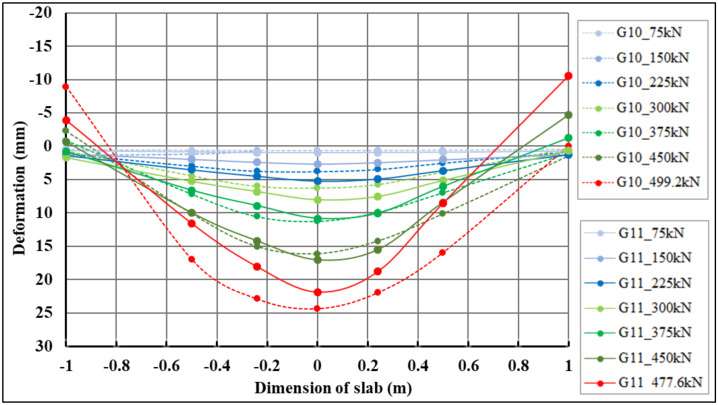
Comparison of 2D cross-deformation sections of centrically loaded slab G10 and eccentrically loaded slab G11 for track sensors 23|23*–29|29*.

Comparative 2D deformation sections at the locations of track sensors 23|23*–29|29*, which depict the behavior of both fiber-reinforced concrete slabs, are shown in Figure 15. Deformation values for selected load steps and selected track sensors 23|23*–29|29* for centrically (G10) and eccentrically (G11) loaded slabs are shown in Table 7.

**Figure 16:**
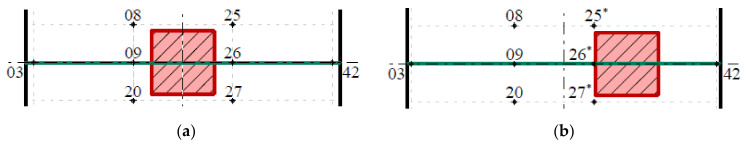
Longitudinal sections: (**a**) centrically loaded slab G10; (**b**) eccentrically loaded slab G11.

In the longitudinal direction, the section corresponding to track sensors 03–42 was evaluated (Figure 16). From the measured data of the scanned track sensors, the deformations of the slabs in the places where the track sensors were mounted were calculated.

**Figure 17:**
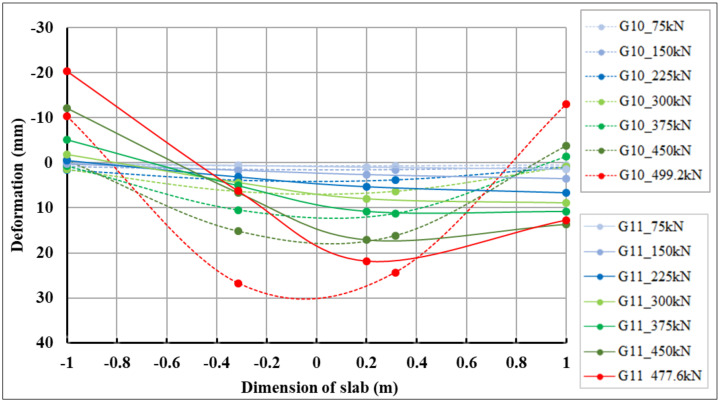
Comparison of 2D longitudinal-deformation sections of centrically loaded slab G10 and eccentrically loaded slab G11 for track sensors 03–42.

The maximum negative deformations in the slabs of track sensors 03–42, which correspond to the lifted corners of the slabs, were 13.06 mm (centrically loaded slab G10, sensor 42) and 20.37 mm (eccentrically loaded slab G11, sensor 03), respectively. The maximum positive deformations in the slabs of track sensors 03–42, which correspond to the pushing of the slabs into the subsoil, were 26.72 mm (centrically loaded slab G10, sensor 09) and 21.84 mm (eccentrically loaded slab G11, sensor 26 *), respectively. Comparative 2D deformation sections in the slabs of track sensors 03–42, which capture the behavior of both fiber-reinforced concrete slabs, are shown in Figure 17. Deformation values for selected loading steps and track sensors in section 03–42 for centrically and eccentrically loaded slabs are shown in Table 8. At track sensor 03, located at the edge of the slab, the lifting of the corner for the maximum load case was twice that of eccentrically loaded slab G11, as is also evident from Figure 17 and Table 8. In the case of the track sensor 42, located on the opposite side of the slab, the deformations were of a different nature. In the case of centrically loaded slab G10, the slab was lifted (negative deformation). In the case of eccentrically loaded slab G11, the slab was pushed into the subsoil (positive deformation), as is evident from Figure 17.

**Figure 18:**
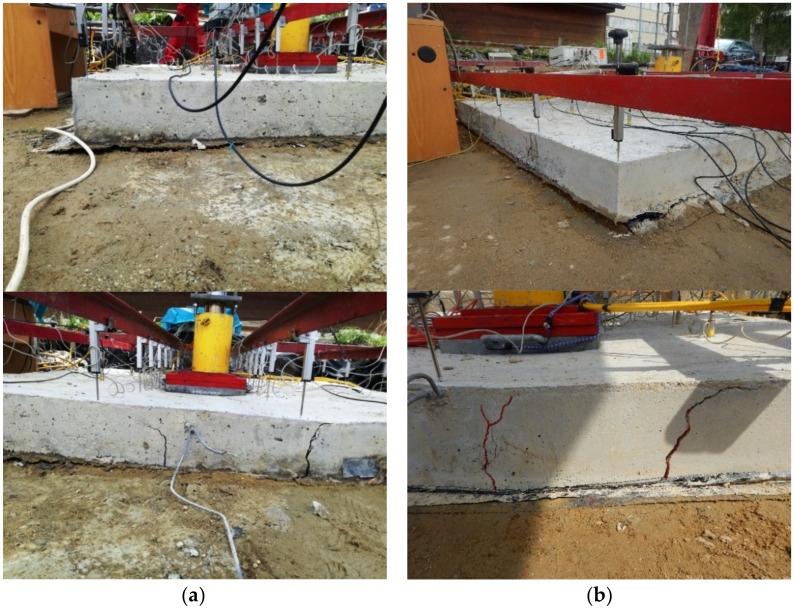
Slab: (**a**) centrically loaded slab G10; (**b**) eccentrically loaded slab G11.

Centrically loaded slab G10 and eccentrically loaded slab G11 during loading are shown in Figure 18. 

**Figure 19:**
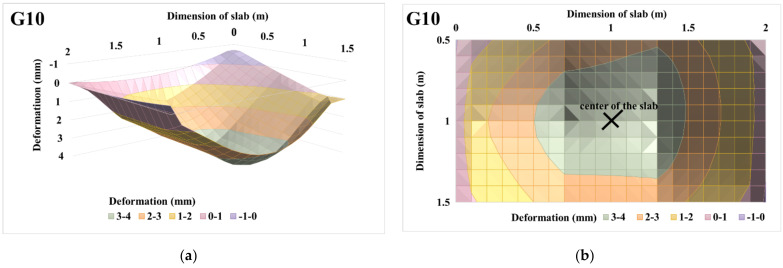
Centrically loaded slab G10 with 225 kN load: (**a**) 3D deformation surface of the slab; (**b**) projection of 3D deformation surface of the slab.

**Figure 20:**
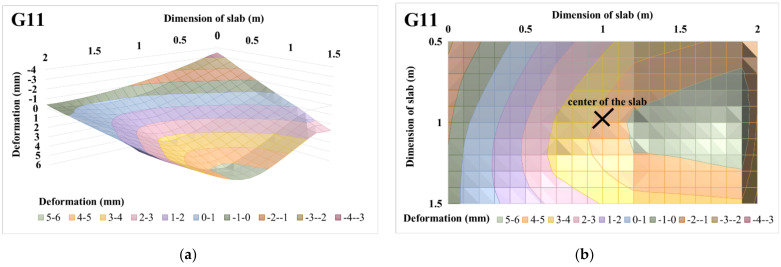
Eccentrically loaded slab G11 with 225 kN load: (**a**) 3D deformation surface of the slab; (**b**) projection of 3D deformation surface of the slab.

The 3D deformation surfaces of centrically loaded slab G10 and eccentrically loaded slab G11 were also evaluated for selected loading steps, i.e., for loading steps of 225 and 450 kN. Part of the evaluation of 3D deformation surfaces was the evaluation of the 3D deformation surface with transfer into the projection, where the maximum deformation area is clearly visible with regard to the applied load and eccentricity. The projections are shown only for the central part of the slab, i.e., for a slab strip of size 1 m. The evaluations of the 3D deformation surfaces of centrically loaded slab G10 and eccentrically loaded slab G11 and the projections of the 3D deformation surfaces for a loading step of 225 kN are shown in Figure 19 and Figure 20. Based on the 3D approximation, the deformations in the center of the slab were determined for a load step of 225 kN. In the case of centrically loaded slab G10, the deformation in the center of the slab was 3.64 mm, while the deformation in the center was 4.26 mm for eccentrically loaded slab G11. Deformations of the slabs at the center of eccentricity were also evaluated for a load step of 225 kN. In the case of centrically loaded slab G10, the deformation in the center of the eccentricity was 3.37 mm, while it was 5.30 mm for eccentrically loaded slab G11.

**Figure 21:**
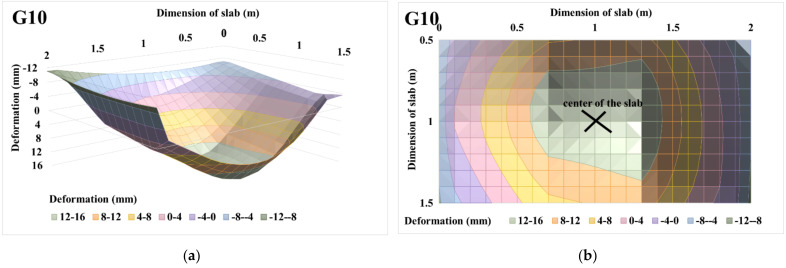
Centrically loaded slab G10 with 450 kN load: (**a**) 3D deformation surface of the slab; (**b**) projection of 3D deformation surface of the slab.

**Figure 22:**
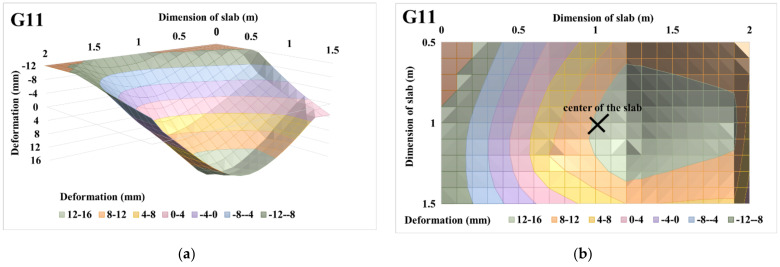
Eccentrically loaded slab G11, with 450 kN load: (**a**) 3D deformation surface of the slab; (**b**) projection of 3D deformation surface of the slab.

The evaluations of the 3D deformation surfaces of centrically loaded slab G10 and eccentrically loaded slab G11 and the projections of the 3D deformation surfaces for a loading step of 450 kN are shown in Figure 21 and Figure 22. Deformations at the center of the slabs and at the center of the eccentricity of the slabs were also evaluated for a load step of 450 kN. In the case of centrically loaded slab G10, the deformation was 14.71 mm at the center of the slab and 13.07 mm at the center of the slab eccentricity. Eccentrically loaded slab G11 had a deformation in the center of the slab with a value of 13.07 mm, and in the center of the eccentricity it had a value of 15.13 mm.

**Figure 23:**
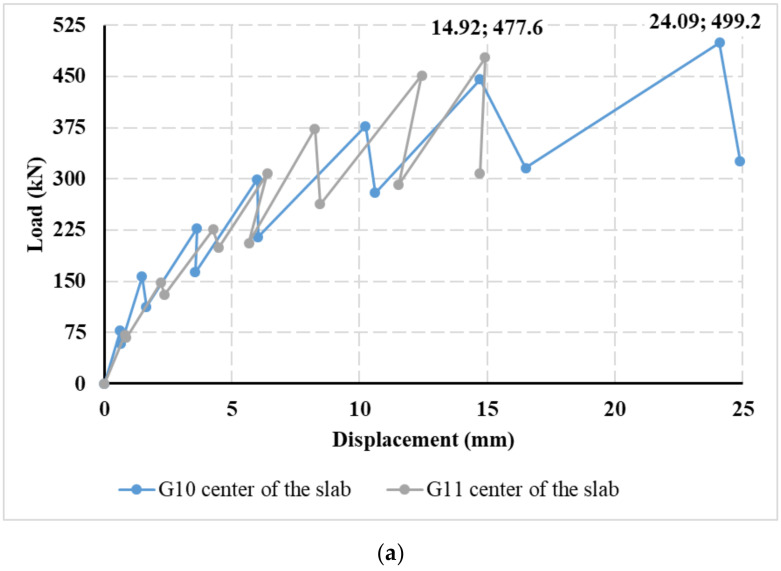

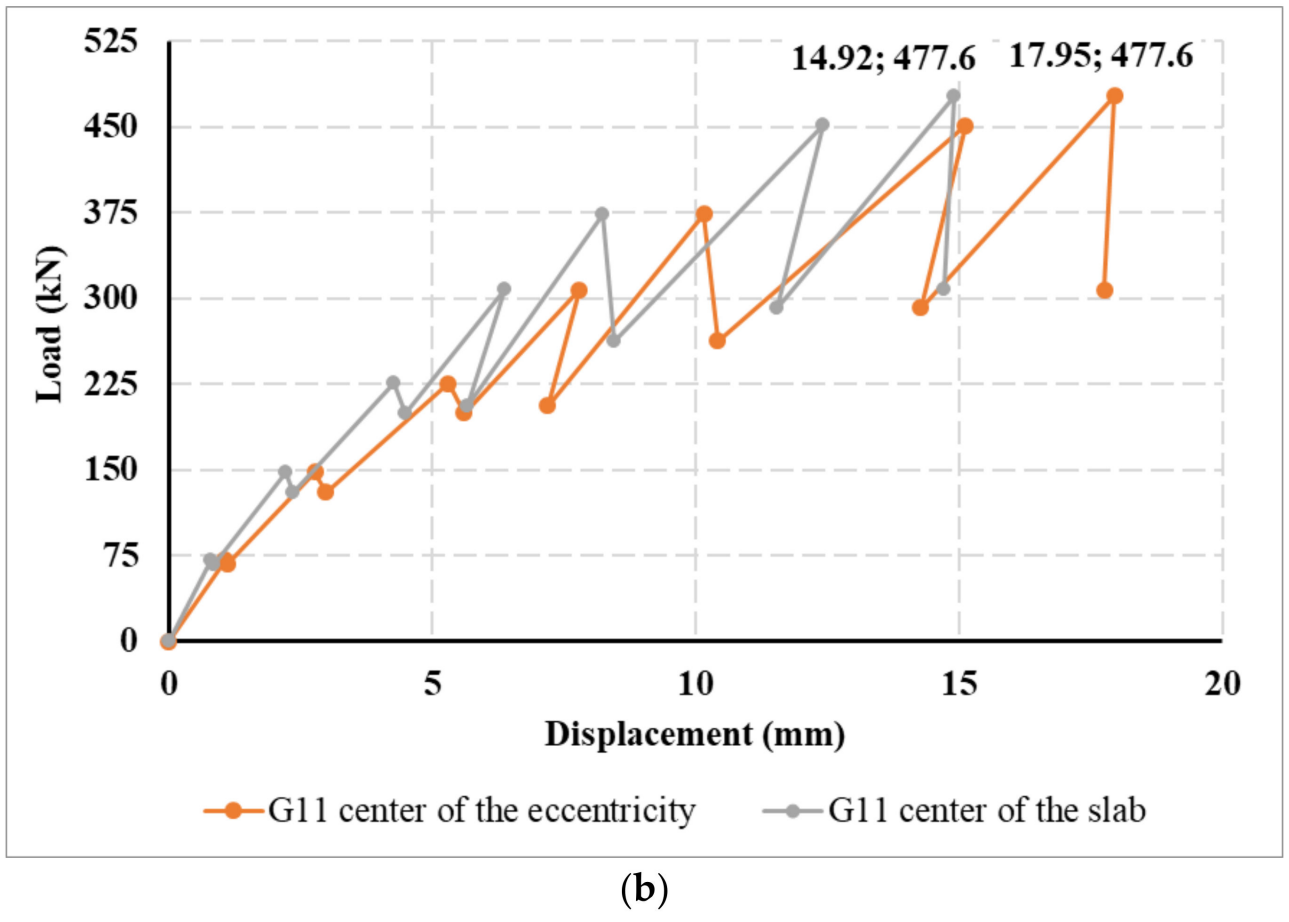
Load–displacement diagrams evaluated on the basis of the approximation of the 3D deformation surface: (**a**) in the center of centrically loaded slab G10 and eccentrically loaded slab G11; (**b**) in the center of the slab and the center of eccentricity of eccentrically loaded slab G11.

Based on the approximation of the 3D deformation surfaces of the slabs, load–displacement diagrams were evaluated, as shown in Figure 23. From the load–displacement diagrams of slabs G10 and G11 in the middle of the slabs in Figure 23a, it can be stated that the course itself is very similar. The resulting deformation in the center of the slab was greater for centrically loaded slab G10. The maximum achieved load for centrically loaded slab G10 was 499.2 kN; this value corresponds to the approximate deformation of 24.09 mm. For the case of eccentrically loaded slab G11, the maximum load was reached at 477.6 kN, which corresponds to a 3D-approximated deformation of 14.92 mm. For eccentrically loaded slab G11, the approximate deformation was larger in the center of eccentricity than in the center of the slab. The approximate deformation in the center of the eccentricity was 17.95 mm, and in the center of the slab, it was 14.92 mm (Figure 23b).

**Figure 24:**
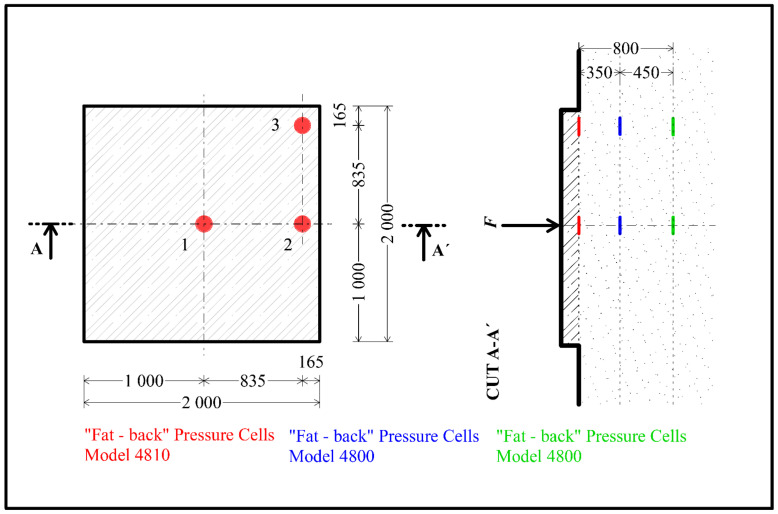
Schema of pressure cells in subsoil for centrically loaded slab G10 and eccentrically loaded slab G11.

Based on the measured pressures in the subsoil during the load test of the slabs, the stresses in three layers at the locations of the stored pressure cells were evaluated. A diagram of the arrangement of the pressure cells is shown in Figure 24. The first layer of pressure cells is located at the location of the foundation joint. The other two layers with pressure cells are located 350 mm and 800 mm below the foundation joint. Three pressure cells are located in each layer. A total of nine pressure cells were located.

**Figure 25:**
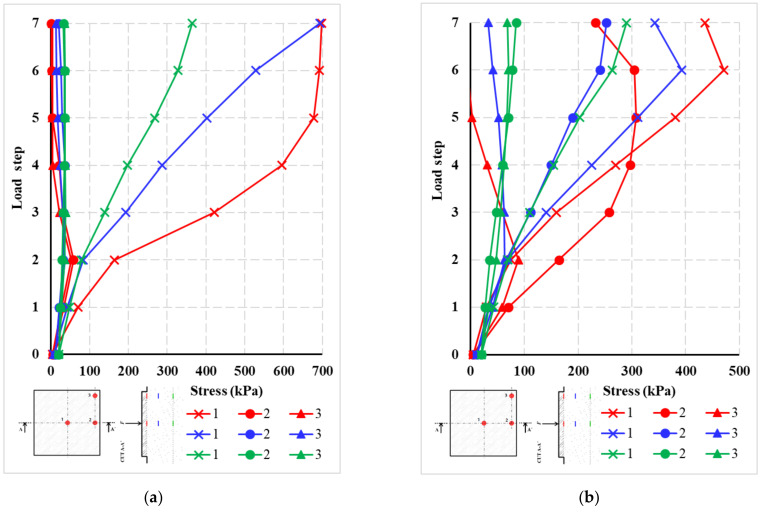
Stress in individual pressure cells: (**a**) centrically loaded slab G10; (**b**) eccentrically loaded slab G11.

Figure 25 shows the stress curves on the pressure cells for each layer. In the case of centrically loaded slab G10, the maximum values were reached for the pressure cells in the center of the slab. The maximum stress in the case of centrically loaded slab G10 reached values of 700 kPa. The effect of the load decreases significantly with the depth of the subsoil. For the deepest point, the stress at the edge of the slab and in the corner of the slab drops to a value corresponding to a maximum of 10% of the stress in the center of the slab. In the case of eccentrically loaded slab G11, measuring points 2 and 3 are closer to the load. For this reason, the measured stresses in the pressure cells are higher compared to centrically loaded slab G10. Again, for pressure cells located deeper, the stresses on the cells are significantly lower. The maximum stress approached 500 kPa.

The individual layers of the pressure cells or the stress curves in the pressure cells are differentiated by color in Figure 25. The color resolution of the individual layers is based on Figure 24. The layers of pressure cells in the foundation joint are marked by a red curve, the second layer of pressure cells (350 mm below the foundation joint) is shown by a blue curve, and the third and final layer of pressure cells (800 mm below the foundation joint) is represented by a green curve.

**Figure 26:**
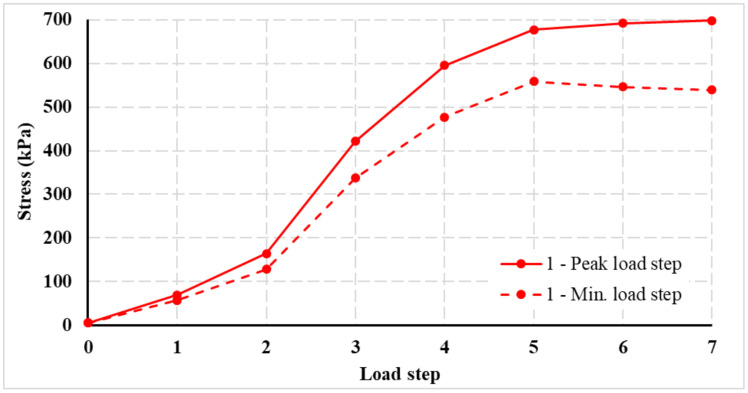
Example of stress for pressure cell 1 in the first layer (foundation joints)—centrically loaded slab G10.

It can be seen from Figure 12 that the loading of the slabs took place in loading steps. Each loading step was 75 kN. After reaching the maximum load in a given loading step, the slab relaxed for 30 min. After this, another loading step followed, and there was time for the slab to relax. This was repeated until the maximum slab load was reached. Figure 26 has two stress curves showing the stress profile of a pressure cell. Figure 26 shows a different course of stress at the time of reaching the maximum load for a given load step (solid line—marked in the graph as peak load step) and at the time of load after 30 min of slab relaxation in a given load step (dashed line—marked in graph as a minimum load step). The stress on pressure cells after the slab relaxation time was lower.

## 6. Discussion

### 6.1. Fiber-Reinforced Concrete Slabs

The laboratory tests and tests of structural elements verified that fiber-reinforced concrete can significantly contribute to the structural and material optimization of the design of concrete structures. The use of fiber-reinforced concrete can significantly simplify the production of concrete structures. The selected dosing of fiber of 25 kg/m^3^ is suitable with regard to concreting technology and financial costs. However, in comparison with previous experiments, it can be stated that the total load-bearing capacity of the fiber-reinforced concrete slab (usually around 500 kN) will typically be less than the traditional solution of a reinforced concrete slab with concrete reinforcement, where the load is up to 700 kN. However, the use of concrete reinforcement is more demanding on concreting and overall financial costs. However, the use of only plain concrete for slabs has the effect of creating a significantly lower load-bearing capacity of the slab than using a fiber-reinforced concrete slab. The collapse of a plain concrete slab is also marked by a significantly quasi-brittle nature.

The use of fiber-reinforced concrete also makes a significant contribution to increasing the ductility of the structure, i.e., to the safety of the structure as compared to the use of plain concrete. A broader evaluation of the presented experiments and tests already performed revealed that the overall load-bearing capacity of the slab in the subsoil interaction is influenced by the mechanical properties of the concrete rather than the subsoil. The mechanical parameters of the subsoil significantly affect the resulting deformations of the slab. These can be differences of up to tens of percentage points. From a design point of view, the experiment performed on a fiber-reinforced concrete slab with an eccentric load showed that the resulting load-bearing capacity was very similar to that of a slab with a centric load. This was mainly due to the fact that the slab remained whole throughout the test. However, the eccentric load significantly affected the course of deformations.

### 6.2. Fiber-Reinforced Concrete

The selected fiber-reinforced concrete variant has a number of advantages over plain concrete. However, the quasi-brittle nature of fiber-reinforced concrete in itself requires a set of laboratory tests of the compressive strength of concrete, tensile strength under splitting, modulus of elasticity, and bending tensile strength. From the results of laboratory tests and a comparison with the available recommendations, we confirmed that it is appropriate to carry out specialized tests rather than using only the recommendations for modulus of elasticity and tensile strength under splitting.

It is clear from the tensile tests that the strengths different may be for test setup. However, the recalculated tensile strengths are relatively similar. In the three-point test, the strength values are slightly higher than in the four-point bending test. This is because the location of the crack is not predetermined in the four-point bending test. In the case of the modulus of elasticity, the results of the tests show a significantly lower modulus of elasticity than in Model Code 1990 [1]. In the splitting tensile test, it was shown that the higher the tensile strength, the higher the bulk density.

### 6.3. Subsoil

Important information also includes knowledge about the subsoil, where our analysis revealed that the subsoil has the character of incoherent soil (approximately 55% sand, 15% gravel, and 30% clay). According to the percentage of individual fractions, this soil can be globally classified as clayey sand. The basic soil parameters are cohesion, *c* (kPa), and angle of internal friction, *φ* (°) 34.4. It is suitable to perform tests on the deformation modulus of soil, where the measurement values a relatively wide variance of measured values from 16 to 58 MPa; the resulting values depend on the actual history of loading and the specific climatic conditions. The mechanical properties of the soil are also often affected by the climate at the surface in particular. It is advisable to repeat this test several times to obtain a wider set of static data.

Simplified analytical/numerical models and experiments, such as symmetry, dimension, etc., can be used for typical cases of a slab in interaction with subsoil. Important aspects of the problem include the need to closely monitor the behavior and parameters of the fiber-reinforced slab and subsoil. In the case of fiber-reinforced concrete slabs, a network of vertical deformation sensors was used. In the case of subsoil, specialized pressure cells were used to measure stress. The comparison of the results allows for a detailed view of the behavior of the fiber-reinforced concrete slabs during a load test. A comparison of the results from the fiber-reinforced concrete slab and the subsoil showed that the initial formation of cracks at the lower surface of the slab were manifested by an increase in the contact stress in the middle of the slab. There was a significant increase in stress in the pressure cell in the middle of the slab closest to the surface. A more detailed study of data from soil pressure sensors in the lower layer (at a depth of 0.8 m) revealed that the stresses are significantly lower compared to the contact stresses at the surface. By approximating the stress results in the pressure cells in the individual layers, it was found that the concerned area of the subsoil and the deformation zone for maximum load is about 2 m deep. The specific values measured at the edges of the slab were significantly lower than below the center of the slab. The differences between the measured values were significant. With the achievement of the maximum load-bearing capacity of the fiber-reinforced concrete slab and the formation of significant cracks in the concrete, the stress in the pressure cells no longer increased and remained at the maximum values. This was due to the fact that there was a significant decrease in the bending stiffness of the slab. The differences in pressure cells in the subsoil for the centrically (G10) and eccentrically (G11) loaded slabs are relatively small. Only in the case of higher loading steps, when the corners of the slab were already being lifted, was it evident that the pressure cell was relieved, while the character itself was slightly different for the centrically (G10) and eccentrically (G11) loaded slabs. This is due to the different geometry and position of the load.

## 7. Conclusions

Research on slabs in interaction with subsoil is very demanding. This is because a number of factors enter into the analysis, which involves the use of advanced testing and diagnostic methods. The selected variant of fiber-reinforced concrete slab has a number of advantages in comparison with concrete and reinforced concrete slabs. However, the quasi-brittle nature of fiber-reinforced concrete itself requires an extensive set of laboratory tests. From the point of view of the design of concrete structures, it is important to monitor the deformations and cracks in the concrete of the fiber-reinforced concrete slab itself. By a more detailed study of the results from 2D deformation sections, it is possible to distinguish when the increase in deformations is no longer elastic and cracks have formed in the concrete. It is also clear from the graph that the deformation increment gradually increases within the loading step and the bending stiffness of the fiber-reinforced concrete slab decreases. The fiber-reinforced concrete slab remained whole in both tests; when the maximum load was reached, the cracks were over the entire thickness of the slab. During the test, the edges of the slab gradually began to rise. When analyzing the deformations of the slab with centric and eccentric load, it is also possible to use 3D graphs, which enable a clearer and more comparable display of deformations.

The results of the research can be presented in partial conclusions:

- The differences in the overall load capacity of slabs with centric and eccentric loads are relatively small. The center of the eccentric load was already outside the core of the cross section.
- For lower load steps, the difference in fiber-reinforced-concrete-slab behavior and deformation was relatively small. The differences began to increase, especially in the second half of the load experiment tests (loading).
- The position of the steel load plate affected the position of the cracks in the slab. For the slab with an eccentric load, the deformations were more pronounced on the load side. In the case of a slab with an eccentric load, there was also a more pronounced lifting of the corner on one side of the slab.
- In comparison with previous experiments, it can be stated that better deformation parameters of the subsoil mainly affected the magnitude of deformations of the slab and the subsoil rather than the overall load-bearing capacity.
- It is advisable to repeat the test several times when determining the deformation modulus of the subsoil and the ratio for the secondary and primary cycle, which are used to determine the deformation capacity of the subsoil. Pressure cells in at least two levels are suitable for determining the active deformation zone of the subsoil. By far, the most important are the pressure cells directly under the load in plan view.
- The laboratory experiments and tests of structural elements confirmed that dosing fibers at 25 kg/m^3^ can significantly contribute to increasing the overall load-bearing capacity and eliminating cracks in the concrete.
- The use of fiber-reinforced concrete requires a more detailed determination of its mechanical properties. Tensile strength is especially important. It is also important to produce load–displacement diagrams, which can be used to identify fracture mechanical parameters.
- From laboratory tests in split tension, it was found that the position of concreting affects the mechanical properties in tension, i.e., the number of fibers along the cross-sectional height varies slightly.
- It is important to perform tests of the modulus of elasticity of concrete, where the values found are significantly different from those in the recommendations. Modulus of elasticity values were lower than those of the recommendations.
- When evaluating slab tests in subsoil interaction, it is appropriate to use 3D deformation graphs and to determine the load–displacement diagram for the entire structural element tests.

The benefits of this research include that a comprehensive set of information and knowledge with a detailed evaluation of laboratory tests and tests of structural elements can be further used for advanced numerical-modeling-based non-linear analysis, where an optimized structure solution is solved.

Among the important and new knowledge of the presented research is that the problem of the slab in the interaction of the subsoil can be further extended by the structural solution of the sliding joint. This area can be followed by laboratory research into the properties of sliding joints in the climate chamber and measurements on real structures of the base slab with a sliding joint on the subsoil. The authors will focus on this area in further research.

## Figures and Tables

**Table 1:** Material properties of concrete for laboratory samples and slabs.

Concrete	Consistency	Cement	Min. Cement Content	Water–Cement Ratio: *v*/*c*	Aggregates 0/4 (Mined)	Aggregates 4/8 (Mined)	Aggregates 8/16 (Mined)	Water	Plasticizer
Quantity per m^3^ of concrete	S3	CEM I 42.5 R	300 kg	0.6	870 kg	150 kg	820 kg	189 l	2.9 l
Quantity in %	-	-	12.9%	-	37.3%	6.4%	35.2%	8.1%	0.1%

**Table 2:** Material properties of Dramix^®^ 3D 65/60 BG.

Fiber shape	Hooked ends
Bundling	Glued
Length (mm)	60
Diameter (mm)	0.9
Aspect ratio	67
Tensile strength (N/mm^2^)	1160
Effect on consistence (s)	8
Effect on strength of concrete (kg/m^3^)	15
Modulus of elasticity (GPa)	200

**Table 3:** Strengths of fiber-reinforced concrete determined from laboratory tests.

Type of Test	Number of Samples (pcs)	Min. (MPa/GPa)	Max. (MPa/GPa)	Average (MPa/GPa)	Coefficient of Variation (%)
Compressive strength—cube	12	21.0	28.3	24.0	11.5
Compressive strength—cylindrical	11	18.6	22.9	20.5	8.2
Splitting tensile strength—perpendicular to the filling direction	12	1.8	2.5	2.2	9.5
Splitting tensile strength—parallel to the filling direction	12	1.4	2.2	1.8	15.0
Modulus of elasticity	5	17.0	23.0	20.3	13.7
Three-point bending test	5	3.5	4.4	4.0	8.0
Four-point bending test	4	3.1	4.1	3.7	13.0

**Table 4:** Modulus of deformation—subsoil.

Modulus of Deformation (MPa)	Place of Measurement	First Load Cycle *E_def,1_*	Second Load Cycle *E_def,2_*
Centrically loaded slab G10—before concreting the slab	*e* = 1.50 m	18.7	58.4
Centrically loaded slab G10—before concreting the slab	*e* = 0.75 m	17.3	42.7
Average	-	18.0	50.55
Eccentrically loaded slab G11—after the test	*e* = 0.50 m	16.6	32.6
Eccentrically loaded slab G11—after the test	*e* = 1.75 m (outside of slab)	18.3	42.8
Average	-	17.45	37.7

**Table 5:** Soil properties.

**Physical Characteristics**			
**Characteristics of Subsoil**	**Sample 1**	**Sample 2**	**Average**
Bulk density of soil in natural storage *γ*_i_ (kN/m^3^)	22.1	21.4	21.75
Bulk density of dried soil *γ_d,i_* (kN/m^3^)	20.1	19.3	19.70
Humidity *w_i_* (%)	10.0	10.9	10.45
Specific gravity of the skeleton using a pycnometer *γ*_s,i_ (kN/m^3^)	25.71		
**Strength characteristics**			
Cohesion *c* (kPa)	0		
Angle of internal friction *φ* (°)	34.4		

**Table 6:** Deformation of centrically loaded slab G10 and eccentrically loaded slab G11 for track sensors 06–22.

Load Step (kN)	Track Sensor				
	06	07	09	21	22
	Deformation (mm) Centrically Loaded Slab G10|Eccentrically Loaded Slab G11				
75	0.42|0.39	0.53|0.51	0.63|0.61	0.53|0.56	0.37|0.46
225	0.47|–0.08	2.29|1.5	3.85|3.07	2.58|2.26	1.05|0.07
375	−2.22|−2.97	5.8|1.02	10.48|5.08	5.44|2.46	−0.1|−4.68
Maximum load	−13.93|−11.86	12.01|−2.06	26.72|6.23	12.01|−1.28	−7.2|−20.01

**Table 7:** Deformation of centrically loaded slab G10 and eccentrically loaded slab G11 for track sensors 23|23*–29|29*.

Load Step (kN)	Track Sensor				
	23|23*	24|24*	26|26*	28|28*	29|29*
	Deformation (mm) Centrically Loaded Slab G10|Eccentrically Loaded Slab G11				
75	0.45|0.66	0.59|0.83	0.66|0.96	0.57|0.89	0.41|0.71
225	1.12|1.37	2.97|3.53	3.79|5.25	2.53|3.65	0.84|1.23
375	−0.85|0.76	7.2|6.59	11.27|10.83	6.98|6.03	1.06|−1.29
Maximum load	−8.93|−3.92	16.96|11.55	24.36|21.84	15.94|8.53	−0.02|−10.57

**Table 8:** Deformation of centrically loaded slab G10 and eccentrically loaded slab G11 for track sensors 03–42.

Load Step (kN)	Track Sensor			
	03	09	26|26*	42
	Deformation (mm) Centrically Loaded Slab G10|Eccentrically Loaded Slab G11			
5	0.41|0.11	0.63|0.61	0.66|0.96	0.48|1.43
225	1.6|−0.57	3.85|3.07	3.79|5.25	1.01|6.60
375	0.72|−5.19	10.48|5.08	11.27|10.83	−1.37|10.86
Maximum load	−10.40|−20.37	26.72|6.23	24.36|21.84	−13.06|12.71

## Data Availability

Data is contained within the article.
